# “My 9 to 5 Job Is Birth Work”: A Case Study of Two Compensation Approaches for Community Doula Care

**DOI:** 10.3390/ijerph182010817

**Published:** 2021-10-14

**Authors:** Anu Manchikanti Gomez, Stephanie Arteaga, Jennet Arcara, Alli Cuentos, Marna Armstead, Renee Mehra, Rachel G. Logan, Andrea V. Jackson, Cassondra J. Marshall

**Affiliations:** 1 Sexual Health and Reproductive Equity Program, School of Social Welfare, University of California, 110 Haviland Hall, MC 7400, Berkeley, CA 94720, USA; s.arteaga@berkeley.edu (S.A.); jarcara@berkeley.edu (J.A.); rachel.logan@berkeley.edu (R.G.L.); 2 SisterWeb San Francisco Community Doula Network, 1912 Keith Street, San Francisco, CA 94124, USA; a.cuentos@sisterweb.org (A.C.); m.armstead@sisterweb.org (M.A.); 3 ACTIONS Program, School of Nursing, University of California, 2 Koret Way, San Francisco, CA 94143, USA; renee.mehra@ucsf.edu; 4 Department of Obstetrics, Gynecology, and Reproductive Sciences, University of California, 2356 Sutter Street, J-140, San Francisco, CA 94115, USA; andrea.jackson@ucsf.edu; 5 School of Public Health, University of California, 2121 Berkeley Way #5302, Berkeley, CA 94720, USA; cassiejm@berkeley.edu

**Keywords:** doula, community doula, birth equity, compensation, health equity, labor conditions, maternal health, pregnancy

## Abstract

With the increased policy emphasis on promoting doula care to advance birth equity in the United States, there is a vital need to identify sustainable and equitable approaches for compensation of community doulas, who serve clients experiencing the greatest barriers to optimal pregnancy-related outcomes. This case study explores two different approaches for compensating doulas (contractor versus hourly employment with benefits) utilized by SisterWeb San Francisco Community Doula Network in San Francisco, California. We conducted qualitative interviews with SisterWeb doulas in 2020 and 2021 and organizational leaders in 2020. Overall, leaders and doulas reported that the contractor approach, in which doulas were paid a flat fee per client, did not adequately compensate doulas, who regularly attend trainings and provide additional support for their clients (e.g., referrals to promote housing and food security). Additionally, this approach did not provide doulas with healthcare benefits, which was especially concerning during the COVID-19 pandemic. As hourly, benefited employees, doulas experienced a greater sense of financial security and wellbeing from receiving consistent pay, compensation for all time worked, and benefits such as health insurance and sick leave, allowing some to dedicate themselves to birth work. Our study suggests that efforts to promote community doula care must integrate structural solutions to provide appropriate compensation and benefits to doulas, simultaneously advancing birth equity and equitable labor conditions for community doulas.

## 1. Introduction

As health policy advocates and policymakers look toward doula care as an intervention to mitigate adverse maternal and infant health outcomes in the United States, examining the sustainability of providing this type of care has become crucial. A doula is a nonclinical birth worker who provides physical, emotional, and informational support to a pregnant person during pregnancy, childbirth, and the postpartum period [1]. Doulas provide critical support for birthing people, working to ensure that their nonmedical needs are met by providing advocacy, advice, assistance, nonjudgmental support, and comfort [2,3]. For example, doulas help birthing people become informed about their care and birthing options and provide emotional reassurance throughout labor [3]. In the postpartum period, doulas provide advice and support around holistic recovery after birth, as well as the transition to parenthood, including newborn care, infant feeding, and parent–infant bonding [2]. Some doulas also provide support for people experiencing abortion, miscarriage, fetal death, and adoption [4]. Importantly, doulas do not provide medical care and therefore cannot replace clinicians.

In the US, doula care is not typically covered by health insurance. A pregnant person may hire a doula privately for an average cost of 1200 USD per birth (ranging from 800 to 2500 USD per birth) [5]. Consequently, private doulas are generally hired by people with the financial resources to afford this out-of-pocket expense [6]. Notably, a 2003 survey of 626 certified and certification-in-process doulas from across the US found that doulas were primarily white, college-educated, higher-income, married women with children who work independently as private doulas [7]. Private doula services typically include one or two prenatal home visits, support during labor and birth, and one or two postpartum home visits, with ongoing communication via text, phone, and/or email [8].

In contrast to private doulas, community doulas are typically trusted members of the communities they serve, sharing culture and language with their clients and often providing more comprehensive services and referrals [6,8]. This shared cultural and linguistic knowledge enables community doulas to better understand the issues faced by their clients, including racial discrimination and language barriers, as well as provide culturally relevant care [6]. Such tailored, intensive support may be particularly effective in mitigating adverse birth outcomes experienced by people of color and those with low incomes. Notably, community doula care is often provided at low or no cost [6]; while this helps increase access to these much-needed services, it also creates barriers for the sustainability of culturally congruent, community-based doula care.

### 1.1. Benefits of Doula Care

The benefits of doula support include improved health outcomes for birthing people and their babies, increased quality of care, and potential cost savings. In a systematic review of randomized controlled trials, birthing people with continuous labor support from a staff member, doula, or person from their social network were more likely to have a spontaneous vaginal birth and shorter labor and less likely to have a cesarean or instrument-assisted delivery, use regional (i.e., epidural) or any pain medication, have a low infant Apgar score, and report negative ratings or feelings about their birthing experience compared to usual care [9]. Additional studies also found lower rates of cesarean sections and preterm birth (<37 completed weeks of gestation) [10,11,12] and higher breastfeeding initiation rates in doula-supported births [13].

The improved outcomes associated with doula support can result in reduced medical expenditures. For example, cost-effectiveness analyses indicate potential cost savings related to lower rates of cesarean deliveries and preterm births if doula services are reimbursed at an average of 986 USD per birth (ranging from 929 to 1047 USD per birth) [12]. Furthermore, estimated state-level cost savings from reductions in cesarean deliveries for doula-supported Medicaid births range from a median of 900,000 USD to 11 million USD annually, depending on doula reimbursement rates [10]. Despite meeting the Triple Aim of maternal care—improved health outcomes, better quality of care, and reduced costs [14]—doula support is not widely available or used in the US. In a representative sample of birthing people in California in 2016, approximately 10% received doula support, while only 6% of a nationally representative sample of birthing people in 2011–2012 received doula support [15,16].

### 1.2. The Role of Community Doulas in Reducing Health Inequities

Birthing people of color experience poorer pregnancy-related and infant health outcomes than white birthing people in the US. Compared to white birthing people, Black birthing people have over three times the risk of maternal mortality [17,18], and Black, Latinx, Indigenous, and Asian/Pacific Islander birthing people have a higher risk of severe maternal morbidity, including cardiovascular disease, cerebrovascular disease, pulmonary disease, sepsis, and shock [19]. Additionally, Black and other birthing people of color experience higher rates of cesarean delivery and adverse birth outcomes, such as infant mortality (death within the first year of life), preterm birth, and low birth weight (<2500 g) [20,21].

The causes of racial inequities in maternal and infant outcomes are multilevel, including factors manifesting at the individual (e.g., socioeconomic status, comorbidities), community (e.g., social networks, civic engagement), healthcare provider (e.g., knowledge, cultural competence, implicit bias), and systemic levels (e.g., access to quality care, structural racism) [22,23,24]. As members of the communities they serve, community doulas possess first-hand knowledge of how implicit bias and structural racism affect healthcare access and quality, allowing them to provide care that is culturally sensitive and relevant [6]. Importantly, one focus group study with Black, Indigenous, and white people with low incomes in Minnesota found that participants perceived doula support as mitigating vulnerabilities to health inequities by empowering them with knowledge and skills, connecting them to resources, promoting a sense of security and calm, facilitating interactions with healthcare providers, and improving their agency by facilitating healthcare providers’ respect of birthing people’s autonomy in informed decision making [25]. Yet doulas may be least accessible to pregnant people from communities most likely to experience adverse outcomes, despite high levels of interest, as evidenced by a representative survey of California women who had recently given birth that found that Black women were more likely to be interested in future doula use than women from other racial and ethnic groups [16]. Barriers to obtaining doula support include cost, particularly for people with low incomes, and limited availability of racially and ethnically diverse doulas [7,10].

### 1.3. Compensation Approaches for Doulas

Doulas may be paid as independent contractors or as employees. The independent contractor approach is typical in private-pay doula care in the US, in which a client pays a flat, out-of-pocket fee for a specific menu of services. Alternatively, doulas may be paid as hourly or salaried employees of an organization; this approach may be more common for community doulas, as care is typically provided at no or low cost to the client. Notably, doula care is not typically reimbursed by third-party payers, such as health insurers [7]. A 2016 survey of 98 existing community doula programs found that 89% of programs paid doulas with salaries; however, sustainable funding of such programs was a concern [26]. Furthermore, 80% of the surveyed community doula programs were run by nonprofit organizations, of which 70% received funding through private foundation grants; less than 5% of programs received reimbursements from Medicaid, a government-funded insurance program for low-income people, despite nearly all (96%) programs serving people insured by Medicaid [26]. Therefore, to ensure sustainability of funding and to serve communities at the greatest risk of birth inequities, community doula programs and birth justice advocates are pursuing Medicaid reimbursement, as well as funding from foundations and state and federal sources [26].

Four US states are implementing Medicaid reimbursement for doula services: Oregon, Minnesota, California, and Indiana [27,28,29,30,31]. Notably, California’s benefit is specifically for community-based doula care and will be implemented starting in 2022 [31], and Indiana’s legislation does not include funding to implement a system for reimbursement [27]. New York is currently piloting a Medicaid reimbursement program for doula care services [32], and several other US states have introduced legislation for such programs [27,33]. Barriers to doulas care being included as a benefit by Medicaid include reimbursement procedures, low reimbursement rates, and, for community doulas in particular, incomplete coverage of the full range of services provided [8]. For example, reimbursement procedures in Oregon and Minnesota require doulas to bill Medicaid independently; thus, community doulas must wait to be paid by Medicaid rather than receive a regular income from their organization [8]. Thus far, Medicaid reimbursement covers a limited number of visits (from 5–9 visits, including labor and delivery), with a global fee-for-service rate of between 350 USD per birth in Oregon to 600 USD per birth in New York [6,8]. In New York, the reimbursement rate was set as a percentage of physician and midwife rates, which does not take into account the significantly longer amount of time doulas spend with their clients (e.g., up to 2 hours per prenatal or postpartum visit) [6]. Moreover, this payment amount would not meet the minimum wage standard for New York City, translating to 5.58 USD per hour after taking into account visit length and frequency, travel time, and remote support [6]. Furthermore, there is no reimbursement beyond the limited number of visits covered by Medicaid, additional time that doulas spend communicating with and working on behalf of clients outside of regular visits or costs related to visiting clients in their homes, including transportation [7,8,28].

### 1.4. Implications for Community Doula Care

With the increased emphasis on promoting doula care as a means to advance birth equity, it is important to determine sustainable and equitable payment models to appropriately compensate community doulas for the care they provide to clients and communities who experience the greatest barriers to optimal pregnancy-related and birth outcomes [6]. Bey et al. (2019) argued that doulas should receive adequate compensation to cover direct services with clients (including both in-person and remote services), administrative responsibilities, training, out-of-pocket expenses, and employee benefits [6]. The purpose of this study was to identify the challenges, benefits, and sustainability of two different payment approaches (contractor with flat-fee per birth and hourly benefited employee) from the perspectives of leaders and doulas in a community-based doula organization in San Francisco, California.

## 2. Materials and Methods

### 2.1. Case Description

Founded in 2018, SisterWeb, a community-based doula organization, works to “dismantle racist healthcare systems, strengthen community resilience, and advance economic justice for birthing families and doulas in San Francisco.” SisterWeb envisions a San Francisco in which “Black, Pacific Islander, and Latina/o/x families… are centered and uplifted in their reproductive journey to welcome their children with respect, dignity, joy, and pride, leading to thriving families and communities and increased birth equity and justice” [34]. In service of this vision, SisterWeb aims to (1) “connect pregnant individuals in San Francisco with culturally compatible birth companions who can help parents-to-be receive the education, resources, and support they need to enter into their birth and parenting journeys with confidence, respect, and dignity,” and (2) “strengthen and diversify the birth provider workforce by training and mentoring birth companions and building relationships with traditional healthcare providers such as physicians, midwives, and hospitals” [34].

SisterWeb provides doula services via three programs: Kindred Birth Companions (KBC), serving Black and African American people; M.A.N.A. Pasefika, serving Pacific Islander communities; and Semilla Sagrada, serving those identifying as Latinx. SisterWeb’s model of care includes three prenatal visits, in-person birth support (when not prohibited due to restrictions related to the COVID-19 pandemic), and four postpartum visits. Additionally, doulas communicate with clients via phone and text message between visits. SisterWeb doulas work in cohorts, consisting of two to three doulas and one doula mentor, allowing them to support one another with the goal of avoiding burnout; the cohort model also allows for the flexibility to consistently provide clients with access to support. Furthermore, SisterWeb doulas participate in regular professional development training and receive time with their mentors to develop their skills as birth workers.

### 2.2. Study Design

Since 2018, SisterWeb and university-based researchers have collaborated to design and conduct process and outcome evaluations. The aim of the process evaluation was to identify the facilitators and barriers to implementation of SisterWeb’s community doula care programs. Our approach diverged from conventional process evaluations in two important ways. First, our research team recognized the highly contextual nature of implementing community doula care programs and that solely focusing on programmatic inputs and outputs in the process evaluation would mask external barriers and facilitators to success. Second, our project drew on the principles of the Equitable Evaluation framework. This framework purports that evaluators have an obligation to contribute to equity, especially when engaged in the evaluation of programs aiming to address inequity, and it stipulates that, for evaluations to advance equity, they must be rooted in collaboration and attend to historical, structural, and cultural contexts [35]. Therefore, our approach extended beyond a typical process evaluation in that we not only examined the internal factors operating within SisterWeb but also the systems and structures in which it operates that influence the organization’s ability to successfully implement programs. Doing so can challenge traditional beliefs and practices about program evaluation that can act as barriers to advancing equity [35]. Throughout the evaluation, we closely collaborated to design and execute evaluation activities, interpret data, and disseminate findings—as evidenced here by the co-authorship of the paper by members of our three organizations. All methods and results are reported according to the Consolidated Criteria for Reporting Qualitative Research [36].

The process evaluation included in-depth interviews with SisterWeb doulas, mentors, organizational leaders, clients, and key stakeholders. Because our participants were known to one another and because power imbalances existed within the sample (e.g., organizational leaders supervising doulas and mentors; stakeholders assisting leadership secure funding and promote the program), focus groups were not an appropriate method of data collection. Individual, in-depth interviews allowed us to gain insights into participants’ true feelings about the SisterWeb program. Additionally, we analyzed programmatic indicator data and collected survey data from two labor and delivery units in local hospitals to understand their preparation for integrating community doulas. Evaluation funding included a focus on the KBCe and M.A.N.A. Pasefika programs; therefore, data from Semilla Sagrada are not included.

In this analysis, we focused on data from SisterWeb doulas interviewed in 2020 (*n* = 6) and 2021 (*n* = 6), as well as SisterWeb leaders (*n* = 4). Given the small number of doulas and leaders, we aimed to interview all potential participants with experience with the two different compensation models. University-based members of the research team, including research staff and graduate students, attended a qualitative interview training led by the first and senior authors and conducted interviews. In line with our participatory approach, two members of the evaluation team presented an overview of the process evaluation to SisterWeb staff in 2019; therefore, two interviewers previously met some participants. We invited all potential interviewees to participate via email.

In early 2020, we invited all nine of SisterWeb’s doulas to participate in in-depth interviews for the process evaluation. Interviews focused on doulas’ motivations for and background in birth work and their experiences being a doula with SisterWeb (including with the organization, clients, the cohort model, their scope of work, and work–life balance and compensation). Interviews with doulas in 2020 lasted an average of 82 min. In early 2021, we invited all eight current SisterWeb doulas who saw clients in 2020 to participate in a second interview focused on doulas’ experiences working with SisterWeb, providing in-person birth support at local hospitals, and how doula work fits into their life, as well as any changes in their experiences and practices since the first interview. Interviews in 2021 lasted an average of 109 min. Lastly, in July 2020, we conducted interviews with all four members of SisterWeb’s leadership circle, which oversees program implementation and administration and acts as SisterWeb’s decision-making body. Interviews focused on SisterWeb’s organizational journey, perceptions of internal and external barriers to program implementation, lessons learned, and adaptations related to the COVID-19 pandemic. Interviews with SisterWeb leaders lasted an average of 95 min. After completing interviews, interviewers took field notes in the form of an interview summary. All interviews were conducted via phone or videoconference, audio-recorded, and professionally transcribed. The Committee for the Protection of Human Subjects at the University of California, Berkeley approved the study protocol.

### 2.3. Data Analysis

We conducted the analysis in two parts. First, we deployed a Rapid Assessment Process (RAP), a team-based, intensive mode of qualitative research that uses iterative analysis, additional data collection, and data triangulation in order to develop preliminary, actionable findings [37]. RAP is well suited for qualitative research informing the design of interventions. Notably, rapid does not mean rushed, and this action-oriented approach utilizes rigorous and strategic data collection and analytic methods to facilitate data reduction and synthesis for time-sensitive projects. For each set of interviews, we developed a summary template with neutral domains matched to interview questions as the first step of data reduction [38]. We then created a summary of each interview using the template. We transferred summary templates to an individual-level data matrix to synthesize key data across participants and domains. Lastly, we created group-level memos to synthesize findings.

Second, we utilized the results of the RAP to develop a parsimonious codebook focused on working conditions for doulas, as key findings from the RAP analysis for the process evaluation were related to the doulas’ experiences with the different compensation approaches [39]. This codebook focused on the rationale, characteristics, barriers and benefits, and sustainability of two of the payment models SisterWeb has used to pay doulas (per-birth, contractor model and hourly, benefited model), as well as overall perceptions about paying doulas for their work and special considerations due to COVID-19. Utilizing a lumping approach, in which larger excerpts of text are coded to identify overall themes, the second author applied these codes to all data from doula and leader interviews using Dedoose, an online, mixed methods analysis program [40]. We then reviewed coded data, memos, and data matrices for emergent themes, comparing the rationale for and sustainability of the two payment models [41]. While we did not employ participant checking, we clarified any questions about program and organizational processes with SisterWeb leadership to obtain a more holistic view of the data.

## 3. Results

Six SisterWeb doulas in 2020 and 2021 and all four SisterWeb leaders agreed to be interviewed; three and two doulas did not respond to invitations to participate in 2020 and 2021, respectively. Five doulas were interviewed at both timepoints, while two doulas were only interviewed once. At both timepoints, we interviewed two M.A.N.A. Pasefika doulas and four KBC doulas. Doulas were between 23 and 38 years old (average age 29 years), and all but two had lived in San Francisco for most of their lives. On average, doulas had been birth workers for nearly 6 years; collectively, at the time of the first interview, they had attended over 135 births. All SisterWeb leaders were either from San Francisco or had lived in the city for over 20 years. All leaders were birth workers, including 3 doulas and a certified professional midwife; they had collectively attended over 800 births.

**Figure 1:**
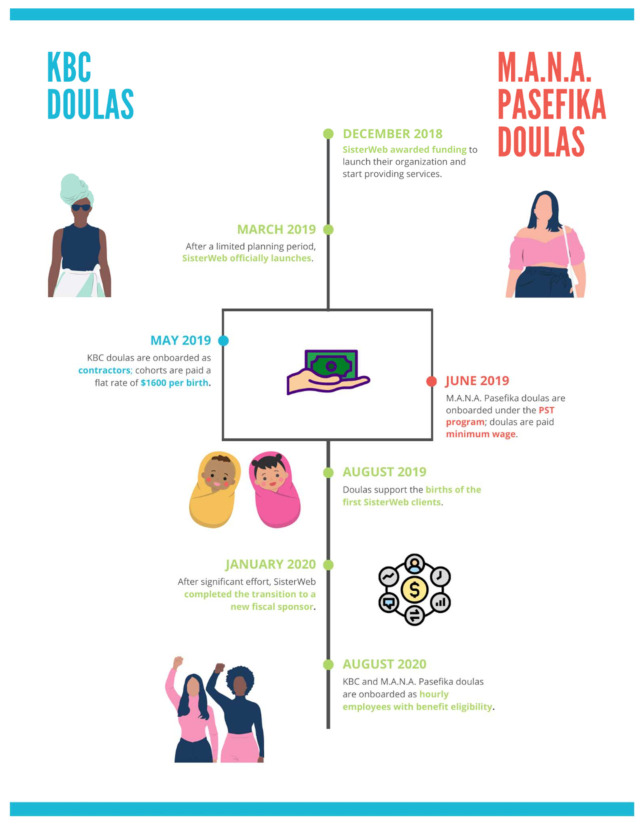
SisterWeb compensation approach evolution.

Two of three compensation approaches utilized by SisterWeb during the evaluation period are the focus of this analysis: (1) initial payment of KBC doulas per birth as contractors and (2) then all doulas as hourly employees with benefits hired by SisterWeb (Figure 1). Due to lack of implementation funding to launch the M.A.N.A Pasefika program, SisterWeb’s Pacific Islander doulas were hired under San Francisco’s Public Service Trainee (PST) program, an entry-level workforce development program that provides temporary employment placements to individuals undergoing job-readiness training. The PST program provided the funding to pay M.A.N.A. Pasefika doulas through the city of San Francisco. Under the PST program, M.A.N.A. Pasefika doulas were paid an hourly rate at minimum wage and were eligible for healthcare benefits. Given that only two doulas were employed under this unique, local program, we do not explore this payment approach in this analysis.

Doulas discussed the benefits and barriers of each model, while leaders described these different models from an organizational perspective, including operations, logistics, and alignment with organizational goals. Both groups spoke about the sustainability of the two models. Below, we first describe experiences with each compensation approach, describing the rationale for and challenges associated with both models.

### 3.1. Independent Contractor Compensation Approach

#### 3.1.1. Rationale

In 2018, SisterWeb was tasked with developing a community doula organization from the ground up very quickly. As SisterWeb is one of the first organizations of its kind, founders had limited examples to follow in developing their programs. With 1 week’s notice to apply for seed funding, SisterWeb founders quickly mobilized to develop and submit a proposal for their community doula organization. The lack of time to engage in thorough research and lack of alternative approaches led them to adopt a compensation approach heavily based on what private doulas and a few similar organizations were offering at the time.

“*We basically—again, with a week’s notice—pulled together a proposal. At that moment in time, we based our doula program a little bit off of what private doulas offer. Also, reading about other community programs… So, we looked at, okay, real quick, what is everybody else doing? We created a mash-up of some of the different elements of their programs and thought—again, without a long, slow process—that was what our best guess was*.”

(Leader)

On the basis of these existing models, SisterWeb developed an initial compensation approach for KBC doulas that consisted of hiring doulas as contractors. Under the contractor payment model, KBC doulas were paid 1600 USD per birth, an amount determined based on compensation rates from other doulas programs and private doulas. This fee was typically split between doulas in a cohort according to workload; cohorts decided for themselves how to manage the workload and compensation balance. While doulas were paid per client, the overall payment amount was intended to be inclusive of their time spent at SisterWeb meetings and professional development training, as stated in doulas’ contracts.

From the beginning, SisterWeb leaders suspected that the contractor compensation model would not be conducive to their goal of providing sustainable, dignified employment for their doulas. However, due to the terms of their initial funding proposal and subsequent contract, they were obligated to use this model to begin providing services and paying their doulas.

“*In the beginning we said, hey, this is how it’s being paid. It’s not really fair. It doesn’t even make sense for us, and we don’t have another system because we have to start working. And we are committed to changing and figuring out a different system, but in order for us to start, we have to start somewhere. If you want to get paid to do this, we have to start somewhere, and this is how we have to start. This is the only option right now.”*

(Leader)

Despite these concerns, SisterWeb launched its KBC program with the contractor, per-birth payment model, with plans to evolve the payment model and provide benefits as soon as it was feasible to do so.

#### 3.1.2. Financial Challenges

SisterWeb leaders and KBC doulas quickly confirmed that the contractor, per-birth model was not sustainable. Notably, this model was not commensurate with the amount of work doulas were doing. Some doulas noted that their workload was highly variable depending on the client; some clients needed extra support and required more contact with their doulas, while SisterWeb’s standard model of care was adequate for other clients. However, doulas would still receive the same amount of pay for every client. When asked if she felt the amount of pay was “enough, too much, or just about right”, one doula responded as follows:

*“It will depend on the client, because there are some that don’t require a lot of the consistent communication. There are some that know exactly what they want you for, and that’s what you’re there for. There are some that don’t know anything and call you… at 4:00 in the morning because they up with cravings and don’t know what to do, or they’re breaking down about something that they can’t control. So, it really just depends on the client.”*

(Doula)

Another doula described the difficulty of having to balance providing her clients extra support beyond what private doulas offer with the fixed amount of pay she receives. When asked how she decides whether to provide extra support for clients or not, one doula said the following:

*“Honestly, if I have the time, like I’m willing to do it. It’s a lot of time and that is one of the drawbacks when you don’t get paid hourly, but like, personally, as long as like, I have availability, I’m willing to go… We kind of like, I guess, base it on urgency of [the] client in the situation. Like, if [a doula] see[s] their client’s really anxious or they’re stressed.”*

(Doula)

Another shortcoming of the contractor, per-birth payment model was doulas’ perceived lack of payment for attending meetings and professional development trainings. Doulas’ contracts specified that their payments for providing care to clients also accounted for their time attending meetings and trainings; however, some doulas were unaware of this and felt they should be compensated more for trainings.

*“I would say that there should be a little more compensation for like the trainings and stuff because we don’t get compensated for that, but we do get paid for the births and the prenatals. So, I guess when you signed up for it, you kinda knew that that’s how it was gonna go, but I would say, I would like some compensation for the trainings as well. Because that’s like extra days that I’m not working at my other job, that I could be making money.”*

(Doula)

This was especially difficult in the months when doulas did not have a birth or had a client drop out.

*“Say my client dropped out. I don’t have a birth this month. I still gotta go to my professional development and put two hours or eight hours, you know, six hours in a training.”*

(Doula)

In these instances, doulas would not receive pay for attending meetings or trainings until they received a payment for their next client.

Despite the challenges described with being paid as contractors, in their initial interviews, doulas felt that the amount of pay they received for births was generally fair. However, most doulas described having to work other jobs to make ends meet. For some doulas, having a second job was generally not an issue, as their other jobs were flexible enough that they could adjust their schedules to attend births as needed. One doula, who was also a childcare provider, said the following:

*“I’m lucky right now, because with my main job, they are very flexible. So, if I have to go to a birth, just need to give them notice. I don’t have to, and I don’t even have to give them that much notice, because the dad at work, he can work from home at any time. He always told me like from the beginning, if anything ever comes up, just call me and we could always like, I can always rearrange my schedule and work from home.”*

(Doula)

However, another doula described difficulties balancing her work with SisterWeb and her other job.

*“I changed jobs toward the end of last year. When I first got, like, interviewed with the job, I was very transparent with them about…what I do on the side. I’m on call, that means, you know, I may have to leave unexpectedly. And the manager at the time was cool with it. But they did change management earlier this year, and he’s not as cool with it, so it’s kinda forcing me to look for another 9 to 5. That’s one of the biggest struggles of it, is just like, finding something where they’re comfortable with what you do on the side.”*

(Doula)

This doula prioritized her work with SisterWeb, but she needed additional employment to ensure financial stability. Therefore, she intended to look for different employment that would be flexible enough for her doula work. Another doula mentioned the financial difficulty of getting to and from client meetings and births without a car.

*“I wish there was some like compensation around like travel. I think that’s my biggest thing right now… especially because Lyft’s and Uber’s like quite expensive, and they add up.”*

(Doula)

Despite the challenges some doulas faced with the contractor compensation approach, they described being happy to receive any payment for doing doula work in their communities.

*“Being able to work with my community has meant everything to me. Because that was like, when I became a doula, that was what I really wanted to focus on, was like working with Black women and people in my neighborhood and stuff like that… it was just hard to be able to work with my community but also like get paid, to be able to live and stay in my community. So, [working with SisterWeb] has been kind of the best of both worlds.”*

(Doula)

*“I love this [work] so much that I would do it for free, but just like… knowing that [working with SisterWeb] can help me a little bit, like, take off the burden of that insecurity, like, that is honestly like a dream come true for me.”*

(Doula)

For most SisterWeb doulas, being compensated for supporting birthing people in their communities was like “a dream come true”, especially given that many community doulas work for free. Being paid as a contractor allowed them to engage in work they loved, even if they had to work other jobs to make ends meet.

#### 3.1.3. Organizational Administrative and Ethical Challenges

In addition to financial challenges, SisterWeb leaders and doulas also identified administrative and ethical challenges with the contractor compensation approach. One major challenge was the multistep administrative process required by SisterWeb’s original fiscal sponsor to issue payments to doulas.

*“We realized that there were major flaws in the program in how doulas were invoicing and getting paid that were just not sustainable. They didn’t align with our ultimate goal of the program, which was dignified employment, to turn birth work into a career. There just wasn’t enough stability. There wasn’t enough guarantee of payment. The timing of the payments was all off.”*

(Leader)

*“The doulas ain’t been paid since December… It’s not affecting them meeting with their clients. It’s not affecting their output. But I definitely see it affecting their state, their emotional state in terms of like, ‘Hey, I put them invoices in. I’m getting emails. Hey, I’m getting the invoices in, when we gonna get paid?’”*

(Doula)

Leaders also described ethical concerns with the contractor model in the context of the COVID-19 pandemic.

*“For SisterWeb, specifically [COVID-19] presented a whole bunch of questions for us around risk and… that we may be asking our doulas to assume a certain amount of risk by going into the hospital during a pandemic when we just didn’t feel comfortable with the amount of financial and healthcare support that we were able to provide them. Where it was, like, for hospital workers, nurses, doctors, midwives—they have salaries and health benefits and extra sick leave.”*

(Leader)

These concerns led to delays in SisterWeb doulas returning to in-person birth support, even after hospitals began allowing doulas to attend births again.

*“One of our biggest pushes in why we weren’t ready to go back into the hospitals is because our doulas were independent contractors. We weren’t sure who had health insurance, and what people’s living situations were like, what their own health was like, their own levels of comfort and safety in working in the environment.”*

(Leader)

Without the ability to provide sufficient financial support and health insurance, SisterWeb leadership did not feel comfortable allowing their doulas to return to in-person birth support, given the potential consequences for their doulas if they contracted COVID-19, including costly healthcare bills, loss of income, and inability to fulfill caretaking responsibilities.

### 3.2. Hourly Employee with Benefits Compensation Approach

#### 3.2.1. Rationale

SisterWeb leaders were always clear about their goal of providing sustainable, dignified employment for their community doulas and, therefore, were committed to transitioning doulas to a compensation approach that better aligned with this goal.

*“Our ultimate goal of the program, [is] dignified employment, to turn birth work into a career.”*

(Leader)

*“We have always been very clear that our doulas get paid to get trained and get paid to do the work that they do. And they are from their own communities, serve their communities.”*

(Leader)

*“Even though this is nonprofit, for me, a for profit would be making sure that my staff would be paid and get the hours that they need and the benefits that they need and that they’re cared for, and they have the support that they need to do the work that they want to do.”*

(Leader)

This goal, as well as the various challenges with and barriers to sustainability of the contractor compensation approach, informed SisterWeb’s decision to transition their doulas to an hourly employment model with benefit eligibility. This transition took longer than expected. First, SisterWeb needed time to raise the necessary funds required to hire doulas as hourly employees.

*“Seeing the numbers play out, and what [it] would cost to put doulas on as staff as employees… I was like, there’s still no way to do it, because if we do that, that shrinks down the budget… I only have a certain dollar amount, and a dollar amount is a dollar amount, and that would shrink my amount to pay our doulas.”*

(Leader)

*“The steps that we had to go through to actually change the model were pretty slow because, for one, we didn’t have the money in the bank to turn everyone into an employee. So, there was an incredible amount of fundraising that had to happen to supplement that initial funding that we got.”*

(Leader)

Additionally, the transition to the hourly employment model coincided with SisterWeb’s transition to a new fiscal sponsor, as their initial fiscal sponsor did not provide them with needed administrative or technical support. The transition required a tremendous amount of administrative effort.

*“We ended up having to switch fiscal sponsors. That, also, was a huge energy and time drain on the organization, especially on the leadership… I would say hundreds of hours that we all spent filling out paperwork.”*

(Leader)

Because the doulas would be employed through SisterWeb’s fiscal sponsor, the transition to the new fiscal sponsor had to occur first.

As of July 2020, all SisterWeb doulas are paid as hourly employees, rather than contractors. Doulas are employed for up to 32 h a week at a regular wage of 25 USD per hour, a wage based on comparable city workforce development programs and doula wages in California. They are eligible for benefits, including health insurance and paid sick leave. Additionally, doulas are paid for all time spent in meetings and training.

#### 3.2.2. Doulas’ Experiences as Benefited, Hourly Employees of SisterWeb

Doulas identified many positive aspects of the benefited, hourly employment model. One of the main advantages mentioned was the ability to rely on a consistent paycheck.

*“I know that I’m going to get paid a certain amount every two weeks. So, I like that. It’s reliable money.”*

(Doula)

*“I like the consistency of the pay now, whereas when we were doing the invoicing, it was whenever you had a birth, you submit it. And then sometimes, I feel like, when we did the invoices, they would take a long time [to process]. So, it was a hit or miss with that.”*

(Doula)

Notably, one doula mentioned how having a consistent paycheck enabled her to have proof of income when necessary, such as when applying for housing. In addition to consistent, reliable pay, doulas described how the hourly employment model ensured they were getting compensated for all of their activities. One doula said the following:

*“Some clients got you working hella extra… So, I think [the hourly model is] more beneficial because you actually get compensated for what you’re doing. And it’s not this shift of you could put in 20 h with one client and get the same little 1600 USD or whatever it was. Or you could put in 5 h and get the same amount.”*

(Doula)

Doulas also valued the benefits that came along with being SisterWeb employees compared to being paid as contractors.

*“We get paid time off, we get sick leave. We get health benefits, dental benefits… beforehand, we didn’t have a lot of those extra perks with being an employee, rather than being an independent contractor. So that’s really good.”*

(Doula)

Another doula mentioned how these benefits extended to her children.

*“I appreciate this [health insurance] because it just has [coverage] for my kids, and emergency stuff, and all of it, just kind of included… so I was able to put them on [my insurance]. And that was a big deal.”*

(Doula)

Notably, all doulas preferred this new hourly employee compensation approach to the models they were paid under previously. However, some doulas mentioned that this new model required more work on their part, as it involved recording their hours more closely.

*“It’s just tricky trying to write down exactly what you do during the day, because I have to write it down on a spreadsheet… Say you talked to your client on the phone for five minutes. And then you talked to your other client for an hour. And then you talked to your other client for seven minutes on the phone. Just trying to figure out, so how much time is that that you spent on that particular work? And then how do you translate that into the spreadsheet? And then how do you translate that into [the timekeeping system], where you log your hours?”*

(Doula)

Importantly, one doula spoke about the difficulty some doulas experienced with transitioning from contractors to employees under the new compensation model.

*“This is a legit job now, going from that shift of being a contractor to then, ‘No, you’re an employee. You need to figure out how you’re getting all these hours done. If I call you during the hours that you’re working, you should be working.’ Because it was so much more free before… Doulas who we’ve hired that come straight into an employee position, they get it.* *But doulas who have gone through all of these changes with us, it’s a lot harder to shift.”*

(Doula)

A few doulas also described minor issues getting onboarded as employees, including learning the new timekeeping system, and lacking knowledge on health insurance options. One doula mentioned that, while she liked the new compensation approach, it still did not address the on-call nature of doula work, which requires being available to attend a birth at any moment, and how to get compensated for this scope of work.

#### 3.2.3. Sustainability

Importantly, the hourly payment model allowed some doulas to devote themselves to birth work and quit other jobs due to the ability to work increased, consistent hours. One doula described the benefits of not having to work other jobs.

*“My favorite thing about working with SisterWeb is that my 9 to 5 job is birth work. It’s like, usually most people have to choose between doing reproductive justice stuff, or having a 9 to 5, where you do both and burn the candle at both ends. And I just love that I’m getting paid a wage that I can with ease afford my rent. With ease buy groceries. I’m not having to scrounge for bills, just because I’m doing birth work.”*

(Doula)

Notably, some doulas were not aware how much time they were spending on their SisterWeb work until they began tracking their time as hourly employees.

*“I never realized how long I was actually sitting there, inputting all of these notes from the birth. I’m like, ‘Oh my God, this takes so much longer than I thought it takes,’ when you’re actually keeping track of it. That and other mundane tasks, like filling out a referral, or texting a client. Or we never realized how what seems like minutes is actually, when you add it all up, it’s hours.”*

(Doula)

When asked if the amount of pay was “not enough, too much, or about right”, most doulas felt that the amount they were getting paid under the new hourly payment model was “about right” or “fair”. However, three doulas noted that, because of the work spent on program development and the cost of living in San Francisco, a higher wage would be beneficial.

*“[A]nother reason [it’s not enough] is just living in San Francisco. Another reason is the added work that we do for program development now. I feel like we’re building our skills. So not only are we doulas, but we’re, like, I would say, community organizers.”*

(Doula)

## 4. Discussion

This analysis highlighted the challenges that a community doula organization faced in realizing its vision of advancing birth equity in San Francisco and community doula care as a sustainable, dignified profession vis-à-vis approaches for compensating doulas. Minimal time for developing a funding proposal and for thorough program planning led SisterWeb to adopt a per-birth, contractor payment model common in private doula care. SisterWeb leaders quickly realized that paying their doulas as independent contractors did not support their goal of providing sustainable employment for their community doulas, who work in cohorts, regularly attend trainings, and often provide additional support for their clients needing more intensive services beyond the scope of fee-based, private doula care models. This is in line with a 2019 report on community doula care models that outlined the many services community doulas provide beyond private doula care, including additional home visits, referrals to social services, engagement with community, and support over a longer period in the postpartum phase [6]. Indeed, SisterWeb doulas reported providing extended care, including attending prenatal care medical appointments with their clients, frequent engagement with clients between visits via phone or text, and helping clients access resources in their communities. Furthermore, the contractor compensation approach meant that doulas did not receive healthcare benefits and undermined organizational goals around community doula care as a sustainable and dignified profession. This was especially significant in the wake of the COVID-19 pandemic, during which attending hospital births in-person posed health risks for doulas—many of whom had other jobs and/or were caregivers who could not afford to be out of work if they became ill. Notably, SisterWeb conducted an internal assessment regarding the impact of COVID-19 after these data were collected; all doulas reported having to use sick leave for COVID-19-related reasons (for themselves or to care for family) and serving as a back-up doula for their peers who had to take such leave [42].

For the community doulas interviewed in this study, there was an evolution in their feelings about compensation approaches. On one hand, in our first round of interviews, doulas generally expressed that they were paid enough and did not particularly mind being paid as contractors; an overall sense of gratitude for the opportunity to do paid birth work pervaded interviews. Notably, one survey of practicing doulas in California found that 55% of respondents provided some services for free, especially when working with clients who lacked the resources to meet their own basic needs [43]; this has also been documented in international settings [44]. For SisterWeb doulas, being compensated to provide services to people in their communities who would otherwise be unable to afford them was a big draw to working with the organization. On the other hand, after SisterWeb transitioned doulas to hourly, benefited employees, doulas expressed a strong preference for this compensation approach. After becoming hourly employees and tracking their time rigorously, doulas realized how much they were working and that they were not getting paid enough before. Furthermore, as SisterWeb employees, doulas experienced a greater sense of financial security from receiving consistent pay, which allowed some to dedicate themselves to birth work, as well as increased health and wellbeing for them and their families via healthcare benefits and sick leave. Importantly, the transition to the hourly employment model was not without its difficulties, both administratively and from the perspectives of doulas themselves. Future research should explore the experiences of doula organizations and doulas with different compensation models, particularly the longer-term retention of community doulas in birth work and approaches for building organizational sustainability. In particular, formal employment may support the professionalization of community doula care, which may have benefits and costs from both organizational and doula perspectives [45].

Doula care is increasingly suggested as a “cost-effective” intervention to improve birth outcomes in the US, particularly for Black women [12,46,47,48,49]. Our analysis suggests that characterizations of cost-effectiveness neglect the costs borne by community doulas, particularly those from and serving communities at greatest risk of adverse birth outcomes. From a numbers perspective, paying doulas as independent contractors may appear more cost-effective, but this effectiveness comes at the expense of adequate compensation of community doulas for their time and for work-related costs such as travel, resources for training, and participation in workforce development activities, and supporting community doula care as a sustainable and dignified profession. While ongoing legislative efforts in numerous states seek to advance doula care by providing reimbursement through Medicaid, these efforts may undermine the feasibility and sustainability of community doula care if they do not ensure a living wage and are based on a medical model of compensation [6,43]. Indeed, an analysis of doula compensation under a Medicaid pilot in New York State found that, for doulas in New York City, the flat-rate reimbursement of 30 USD per visit resulted in an hourly wage of 5.58 USD, after taking into account average travel time and the cost of benefits [6].

Strengths of this study include longitudinal, qualitative data collected from doulas through a long-term academic-community partnership. Through the partnership, the researchers intimately observed the evolution of the compensation approaches and organizational struggles in real-time, allowing for rich data collection that surfaced the nuances of the different payment models. With the longitudinal approach, we completed analysis of data from the leadership circle and the first round of doula interviews prior to developing the interview guide for the second round of doula interviews. As such, our analysis represented iterative data collection and confirmation and evolution of initial findings. Additionally, our sample included interviews with all leadership circle members and most doulas in the two programs included in the evaluation. Limitations of this analysis include the specificity of the geographic context (i.e., the expensive San Francisco, California area), which has implications for the amount of compensation. Future research should investigate the longer-term sustainability of the hourly benefited compensation approach, both for community-based organizations and for doulas themselves.

## 5. Conclusions

For provision of community doula care to be a health equity strategy, community doulas—who, by definition, are community members and often identify as people of color—must have equitable labor conditions. That is, health equity cannot be advanced if intervention strategies rely on community doulas from Black and Brown communities to be contingent, low-wage workers. This was especially pertinent during the COVID-19 pandemic, when providing in-person birth support required community doulas to put their own health at risk; for contractors, this risk occurs without organizational support for health insurance, paid sick leave, and other benefits. As policymakers and private funders seek to promote community doula care, sustainable and equitable compensation models must also be advanced, as doulas may leave the profession due to burnout, low-pay, or lack of full-time employment opportunities. Equitable labor conditions for community doulas may also increase the pipeline of racially and culturally concordant healthcare providers serving pregnant people of color, as doulas may seek opportunities to become midwives, nurses, and physicians. Furthermore, expanding services requires sufficient resources for baseline and ongoing training, as well as workforce development initiatives. Efforts to advance community doula care must integrate structural solutions to provide appropriate compensation to doulas for the full range of their activities to ensure that the benefits of doula care are realized for birth equity and to support equitable labor conditions for community doulas.

## Data Availability

Data are not publicly available due to the risk of deductive disclosure with the case study design and small sample size.
